# Statistical Analysis-Based Error Models for the Microsoft Kinect^™^ Depth Sensor

**DOI:** 10.3390/s140917430

**Published:** 2014-09-18

**Authors:** Benjamin Choo, Michael Landau, Michael DeVore, Peter A. Beling

**Affiliations:** 1 Department of Systems and Information Engineering, University of Virginia, Charlottesville, VA 22904, USA; E-Mails: mjl5b@virginia.edu (M.L.); pb3a@virginia.edu (P.A.B.); 2 Barron Associates, Charlottesville, VA 22901, USA; E-Mail: md9c@virginia.edu

**Keywords:** Kinect^™^, noise model, statistical noise analysis, calibration

## Abstract

The stochastic error characteristics of the Kinect sensing device are presented for each axis direction. Depth (*z*) directional error is measured using a flat surface, and horizontal (*x*) and vertical (*y*) errors are measured using a novel 3D checkerboard. Results show that the stochastic nature of the Kinect measurement error is affected mostly by the depth at which the object being sensed is located, though radial factors must be considered, as well. Measurement and statistics-based models are presented for the stochastic error in each axis direction, which are based on the location and depth value of empirical data measured for each pixel across the entire field of view. The resulting models are compared against existing Kinect error models, and through these comparisons, the proposed model is shown to be a more sophisticated and precise characterization of the Kinect error distributions.

## 1. Introduction

The Microsoft Kinect^™^ is a gesture-based game controller created for home entertainment systems. The depth sensing capabilities of the device, however, engender a number of alternative uses in analysis and control. In [1], for example, the Kinect is used to observe work processes of different workers in a manufacturing cell, with depth data from the sensor used as input for machine learning algorithms that infer worker activity. In robot vision, Cho *et al.* [2] use Kinect as an aide for a human-robot interface, and Susperrigi *et al.* [3] use Kinect in conjunction with a thermal sensor to improve the detection of human presence. Rapid 3D modeling of objects is also benefiting from the use of the Kinect. Nock *et al.* [4] and Azzari *et al.* [5] each present a system utilizing the Kinect for rapid 3D structure measurement of a living plant. Other areas of Kinect's application include aiding hand gesture recognition [6] and improving the detection rate of dangerous events in safety-related systems [7], both of which rely on a precise representation of humans, objects and the background in 3D. An accurate Kinect error model is also crucial to certain object detection and pose recognition algorithms [8–10]. This is especially true when a high degree of accuracy is required and achievable for for objects that are symmetrical, identical to other objects and have small feature sizes within a noisy observed point cloud. Since the Kinect error distribution is not isotropic and an object can be sensed in any region of the field of view, there is the possibility to thoroughly examine the error in the depth images.

Because the Kinect device is widely applied and used in research, numerous scientific reports have been published that attempt to model the systematic lateral and axial error distributions generated from the measured 3D point clouds. For instance, Khoshelham *et al.* [11] and Maimone *et al.* [12] examine the accuracy and resolution of the Kinect sensor in order to correctly calibrate the depth measurements. In their analyses, it is shown that depth measurement error increases quadratically for objects sensed at greater distances. Khoshelham provides a theoretical analysis of why this quadratic relationship is present based on the information from the original Kinect sensor patent [13]. However, Khoshelham's model only uses the depth value to generate the model and does not consider pixel location in determining the error. In other words, a single value is assigned to all pixels across the field of view at a given depth, separately for each of their three *x, y* and *z* individual error models. Menna *et al.* [14] also investigated Kinect's depth measurement performance and describe the basic theoretical workings of the Kinect based on a traditional camera-projector structure. Again, Menna *et al.* disregard the pixel location in building their model and furthermore consider the *x* and *y* directions to have the same error distributions. Chow *et al.* [15] also suggest a bundle calibration approach, as well as a description of the error and noise involved with the Kinect sensor, which is supported by simulation and sample measurement results. However, their simulated result on *σ_z_* is not fitted to an equation, and details on the exact structure of the error model are missing. Miller *et al.* [16] suggest an unsupervised self-calibrating and position locating method for the Kinect that takes into account the distortions in measurement towards the edge of the field of view. Several other calibration techniques have also been reported for the Kinect (see, e.g., [17–19]).

Standards, such as the VDI/VDE 2634 [20], and theory-based error models for *σ_x,y_* also exist. However, the methods employed in [20] are not completely aligned with real-world situations. For instance, a generic sphere placed near the center of the focal plane was used as the test object with suggested sample measurement points in the field of view. In general, though, objects may have more interesting features, such as edges or corners, which may be also sensed closer to the corners of the field of view. Nguyen *et al.* [21] propose another Kinect sensor error model that includes an error distribution for the entire field of view. The model in [21] shows how the Kinect measurements react to tilted surfaces, but does not distinguish between horizontal (*x*) and vertical (*y*) directional errors, reporting instead a single lateral error distribution as a function of the angle formed between the surface and the *z*-axis. Furthermore, the model in [21] is based on measurements only from the center of the field of view. Based on these existing works, a more precise and thorough investigation into Kinect's depth observation is needed in order to accurately reconstruct and model the error distribution that can be applied to the entire field of view.

This paper presents an analysis of the horizontal and vertical stochastic error distributions, as well as the depth directional error distribution of the Kinect sensor based on measurements using a novel 3D checkerboard and flat surface, respectively. As opposed to some previously reported error model suites, we present our three separate directional models, since it cannot be assumed that any two dimensions are correlated. The rest of the paper is structured as follows: In Section 2 the processing internals on the Kinect and the error involved with the device are presented. In Section 3, the measurement setup used in this paper is explained. Section 4 presents results for the flat wall and checkerboard measured data, which is then followed by an analysis and discussion on the proposed model with a comparison to existing models in Section 5. Finally, the paper concludes in Section 6 with remarks on the significance of the proposed method and model.

## 2. Kinect Model and Processing Internals

It is helpful to structure a discussion of error modeling in terms of the model domain, ground-truth domain and measurement domain. The model domain refers to the theory-based model that is used to set up a measurement scenario. This is an ideal, digital domain where all of the lines are straight, all planes are absolutely flat and the dimensions of objects are exactly as designed. The ground-truth domain refers to the actual set up of a measurement scenario. Although effort is made to construct experiments that match the ideal model, the ground-truth and model domains will differ at least somewhat in any experiment, because of limitations on the precision by which calibration targets can be manufactured and measured. Thus, the pursuit of ground truth becomes more of a philosophical question than an actual measurement issue [22]. Finally, the measurement domain is a domain based on the measurement results from the sensor. Therefore, this domain includes sensor error and noise, where sensor error and noise have inherent characteristics from which error models are built.

In order to build an error model, the ground truth of a measured object must be known first. The error component is extracted by subtracting the ground truth from the measured data. The assumption is that error is present in all three perpendicular directions in space, and this error may show different statistical characteristics for each pixel location and for objects at different depths. The goal of the analysis is to characterize this error statistically.

### 2.1. Kinect Camera Model

**Figure 1.**
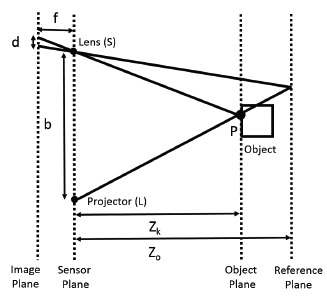
Kinect depth determination model.

The Kinect in a general sense is considered to be a projector-camera-based stereo vision system. This type of 3D vision system uses triangulation to determine the distance from an object to the sensor [11]. A visual top-down representation of the Kinect sensor recording an object via triangulation is presented in Figure 1. Here, the distance between the projector and camera, also referred to as the baseline, is represented with *b*, and the focal length *f* is assumed to be known. The projector emits a structured light pattern within the sensor's field of view, and when an object is present, part of the projected pattern becomes visible to the camera. By matching a segment of the pattern with the point at which it is observed in the camera's image sensor, triangulation is used to determine the distance *Z_k_*. The key is to determine the correspondence between parts of the pattern and its location in the image plane of the camera as the pattern is observed. By the similarity of triangles and the displacement *d* of a pattern from a known reference plane, the distance *Z_k_* of the object from the Kinect sensor can be estimated by:

(1) $$Z_{k}=\frac{Z_{o}}{1+\frac{Z_{o}}{fb}d}$$

The novelty of the Kinect device is that a new structured light pattern and analysis method is proposed that lets the device rapidly determine the correspondence between the projector and camera [13]. The pattern generated by Kinect is designed in a specific way, so that characteristics, such as gray scale, frequency domain peaks and pattern correlation, are highly uncorrelated. In other words, the pattern is distinctively different in a small region compared to neighboring areas, which reduces the possibility for confusion and increases the speed of pattern matching. Furthermore, the Kinect has a built-in database of pattern distortion quantized depth points to further speed up the measurement generation. However, any additional steps before or after triangulation, such as filtering, blurring, cropping, or other exact details on the inner workings of the Kinect device are kept as trade secrets. In the end, a depth reading for each pixel in the 640 × 480 depth image is delivered to the end user.

The origin of the coordinate system used to construct our error models, referred to as the sensor coordinate system, is positioned between the IR camera lens and the projector with an offset of −0.063 mm in *x* and −0.039 mm in *y*, as suggested in [11]. The three axes used in the sensor coordinate system are based on a right-handed notation with the positive *z* direction extending outwards and perpendicular to the IR camera and projector lenses. The horizontal or *x*-axis is defined as the direction parallel to the bottom or top edges of the pixels within the Kinect depth image, with the positive direction extending out towards the right side of the sensor. The *y*-axis is parallel to the side edges of the pixels within the Kinect depth image, with the positive direction extending downwards from the sensor. With the coordinate system defined, the device's raw depth image can be further explained as the collection of depth measurements at each pixel, where the depth represents the shortest perpendicular distance from an object being detected in a pixel's path to the *xy*-plane at the origin. Three parameters with respect to the sensor coordinate system are then sufficient to describe the error distributions associated with the triangulating Kinect sensor. These parameters include the pixel location representing lens angle from the center of the focal plane with respect to the *x*- and *y*-axes of the sensor coordinate system and the depth value of the pixel. This assumption is valid, because we cannot isolate a fourth index of projector angle without changing one of the three other parameters. In other words, referring to Figure 1, we can only change the projector angle by one of two ways: by either keeping the lens angle constant and moving the object along the lens ray pointing to the object (which would change the depth of the object), or by keeping the depth constant and moving the object along the object plane (which would change the lens angle). Therefore, the projector angle parameter is already intrinsically built into our models, thus accounting for errors in triangulation.

### 2.2. Kinect Error Model

**Figure 2.**
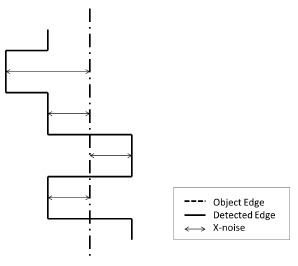
Example of *x*-noise.

Since we treat each dimension of the sensor coordinate system as a unique set of measurements, three models are presented to describe the complete Kinect error distribution. In the first dimension, the axial or depth *z*-axis, error in depth measurements from each pixel are treated as separate values, where the variance in each is used to construct the model. For the other two lateral dimensions, the horizontal and vertical or the *x*- and *y*-axes, it is ideal to define a model with a granularity to cover each of the 640 × 480 pixels. However, for simplicity and practicality, the field of view is divided into 8 × 8 regions, each with a size of 80 × 60 pixels. The error distributions are defined for each region. Horizontal or *x*-axis error is the error generated when detecting an object's vertical edge. In the model domain, a vertical edge of an object appears as a straight line. However, in the measurement domain, the vertical edge of an object appears as a jagged line, as shown in Figure 2. Similarly, the vertical or *y*-axis error is generated when detecting a horizontal edge of an object. Once again, in the model domain, this appears as a straight line, yet in the measurement domain, this line appears jagged. This mechanism differs from other models, where the lateral axes are either treated as possessing the same distributions based only on the pixel radial angle from the center of the focal plane or each pixel within a depth level is treated as an indistinguishable value.

In the model domain, the depth measurement *z* (units of mm) is accurate and the *x-y* coordinates (units of mm) for a given pixel with pixel location *i* – th row, *j* – th column (*i, j*) is determined using the following equations:

(2a) $$x_{I}\left(i,j,z_{I}\right)=P_{h}\left(j-N_{h}/2\right)z_{I}$$

(2b) $$y_{I}\left(i,j,z_{I}\right)=P_{v}\left(i-N_{v}/2\right)z_{I}$$

where *N_h_* and *N_v_* denote the number of horizontal and vertical pixels in the field of view (assumed to be even integers), and *P_h_* and *P_v_* represent the width and height of a single pixel at distance of 1 *mm* from the Kinect, respectively. The quantities *P_h_* and *P_v_* are computed as:

(3a) $$P_{h}=\frac{1}{2}N_{h}\tan\left(\theta_{h}\right)$$

(3b) $$P_{v}=\frac{1}{2}N_{v}\tan\left(\theta_{v}\right)$$

where *θ_h_* and *θ_v_* are the horizontal and vertical viewing angles of the Kinect, respectively 57.5° and 43.5° [23]. Notice that the *x* and *y* coordinates are determined by the depth value *z* and pixel locations *i* and *j.* The center point of a rectangle covered by a pixel was chosen as the point of reference for conversion. In the model domain, there is no consideration for error. This means that all pixels in the field of view correspond to an equally-sized rectangle when projected to a flat surface perpendicular to the *z*-axis. The distance between the centers of each pixel are equal for a given horizontal or vertical direction, as well. This does not mean that the angles at which each pixel are aimed are of equal intervals. Instead, on a flat surface perpendicular to the *z*-axis, each pixel equates to covering the same-sized rectangular area.

**Figure 3.**
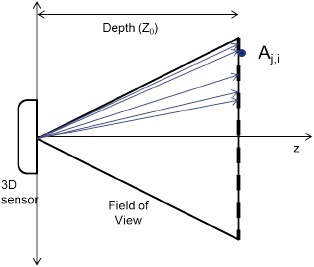
Uneven pixel directions in the real world.

In the ground-truth domain, however, the direction of each pixel may not be correctly aligned, as shown in Figure 3. Each pixel may cover slightly differently-sized rectangular areas. Since the model domain is built under the assumption that there is no distortion in the direction vector, this misalignment or distortion is a source of error introduced into the ground truth in depth *z_G_*. Thus, the *x-y* coordinates must be modified to take into account the error. This error is represented for each of the *z-, x*- and *y*-axes as *∊_z_G__* (*i, j, z_I_*), *∊_x_G__* (*i, j, z_I_*) and *∊_y_G__* (*i, j, z_I_*), respectively. At this point, *∊* (*i, j, z_I_*) is considered to be a constant fixed value without any stochastic components. With the ground-truth vector **G** denoting the vector {*x_G_,y_G_,z_G_*}, the ideal model vector **I** denoting {*x_I_,y_I_,z_I_*} and the error in ground truth ***∊_G_*** denoting {*∊_x_G_,_ε_y_G__∊_z_G__* } in the ground-truth domain, the *z, x* and *y* values of a measured point's coordinates in the sensor coordinate system are represented as follows:

(4) $$\text{G}=\text{I}+{∊}_{\text{G}}$$

In the measurement domain, the distance measurement *z_M_* contains stochastic noise that was not present in the ground-truth domain. In any attempt to measure the ground truth G with sensors, there will always be noise introduced during the measurement process, making it difficult to directly obtain the value of **G**, and so the measurement **M** is used instead. This noise arises from stochastic flickering of IR dots during the correspondence stage, due to thermal changes or fluctuation in the supply voltage. Thus, the noise involved in the measurement domain is represented as a Gaussian distribution, and the distance measurement with error is represented as:

(5a) $$\text{M}=\text{I}+{∊}_{\text{M}}$$

(5b) $${∊}_{\text{M}}=\text{f}\left(\text{I},\mu ,\sigma \right)$$

(5c) μ=E[M−I]

(5d) $$\sigma^{2}=Var\left[\text{M}-\text{I}\right]$$

where **M** is the *x, y, z* coordinates of measured points and **∊*_M_*** is the error represented as a distribution with means ***μ*** and variances ***σ****^2^*, which are different for each dimension. In experiments such as this, it is difficult to establish an accurate ground truth, since it is challenging to establish corresponding model points. Thus, instead of the actual model point stated in the previous equations, the best estimates are used to find the estimated error distribution.

## 3. Measurement Scenarios

This section describes the methodology for measuring the three directional error components. The methodology makes use of two measurement scenarios: the flat surface measurement and 3D checkerboard measurement scenarios. The flat measurement scenario is used to sense a flat surface at known distance intervals, which allows for an analysis of the *z*-axis component of the sensor error. The 3D measurement scenario is used to measure a 3D checkerboard across the field of view at known distance intervals, which allows for the analysis of the *x*- and *y*-axis error components.

### 3.1. Flat Surface Measurement for *z* Error

**Figure 4.**
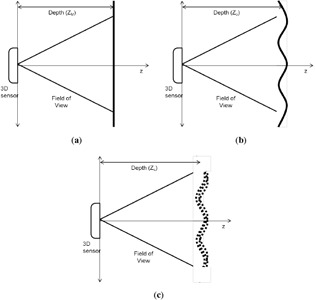
Flat surfaces in the (**a**) model, (**b**) ground-truth and (**c**) measurement domains are displayed.

For determining depth directional error, the flat surface measurement scenario is used. A flat surface is placed at a known distance from the sensor perpendicular to the *z*-axis. In the model domain, the plane is flat and placed at an exact distance from the origin, which is perpendicular to the *z*-axis, as in Figure 4a. The equation for an arbitrary plane in 3D space is given as:

(6) $$z=p_{00}+p_{10}x+p_{01}y$$

where *p*_00_, *p*_10_ and *p*_00_ are coefficients.

In reality, it is difficult to meet the conditions suggested in the model domain. The flat surface may have small, unnoticeable lumps, and the distance from the Kinect to the plane will be slightly different from what was intended. Moreover, the orientation of the plane may not be exactly perpendicular to the *z*-axis. Still, the plane is at some particular distance from the Kinect and in a certain orientation to the *z*-axis. This establishes the ground-truth domain for the flat surface scenario, as shown in Figure 4b. The Kinect sensor then measures the setup of the ground-truth domain and reports a raw data stream of depth measurements with error. The domain built on this noisy sensor data is the measurement domain for the flat surface, as depicted in Figure 4c. The main point of interest is the error that is observed in this domain. In order to determine the error within each pixel observation, information about the ground truth is needed.

The exact figures for the ground-truth domain are unknown. This is because, in order to obtain the ground truth, measurements have to be made using a sensor that itself will be of limited precision. The only information that is available is the model and the measurements, but by fitting a model based on the measurements, a close-to-ground-truth estimate can be made. Though this estimate may not be exactly the ground truth, by controlling the elements of the experiment, an estimate close to the ground truth may be obtained. These elements include finding a flat surface to within several millimeters of unevenness, placing the Kinect to within several millimeters of the intended distance and placing the Kinect as perpendicular as possible to the flat surface. The idea is to make the most intelligent estimation of the ground truth based on the limited information given from the model domain and the measurement domain. In this case, a least squares estimation is used to fit the planar model to the measured data.

Once the ground truth has been estimated, the next step is to compare the measurement against the estimated ground truth. The difference between the estimated ground truth and the measurement is considered to be error. Since, for this paper, the error is considered to have a stochastic component from sensor noise, the error models should be represented as probability density functions or histograms for every pixel at every depth. For the flat surface, measurements were made at 200-*mm* intervals, starting from a distance of 800 *mm* from the sensor and ending at a distance of 3000 *mm* from the sensor.

### 3.2. 3D Checkerboard Measurement for *x* and *y* Error

**Figure 5.**
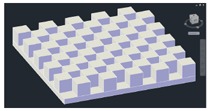
3D checkerboard design.

The best way to determine the *x* and *y* error would be to build differently-sized cubes that exactly fit the size of one pixel at different distances from the Kinect and to move the cube through all of the pixel locations. However, there is significant difficulty in executing such an experiment. Instead, in this paper, the use of a 3D checkerboard is proposed. This checkerboard has boxes placed in a checker pattern, as in Figure 5. The 3D checkerboard has multiple indentations, allowing for a distinct pattern to emerge in the depth image. Furthermore, the edges of the cubes are aligned, and the edge lines intersect at 90 degrees. This allows for the analysis of both *x*- and *y*-axis error from a single depth image.

**Figure 6.**
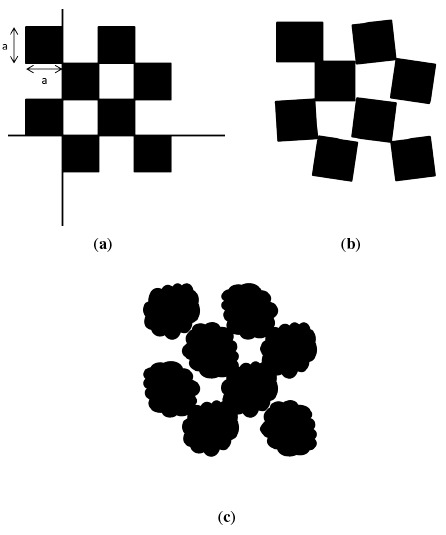
3D checkerboards with sides of length a in the (**a**) model, (**b**) ground-truth and (c) measurement domains are displayed.

Once again, in the model domain, the checkerboard is assumed to be of exact dimensions with the edges aligning to a straight line, as shown in Figure 6a. As in the case of the flat surface scenario, the 3D checkerboard in the ground-truth domain will not match the design of its counterpart in the model domain. Inaccuracies introduced during the manufacturing process of the 3D checkerboard result in issues, such as the edges not being aligned properly or the length of the edges not being exact. An exaggerated example is illustrated in Figure 6b. The Kinect sensor measures this checkerboard and returns a raw data stream with error, as illustrated in Figure 6c.

The model of the checkerboard is then fitted against measured data. Once the model is fitted, the distance to the closest horizontal or vertical model line is calculated for each of the edge points in the depth image, as in Figure 2. If the closest line is a horizontal line, the error is categorized as *y* error, and if it is a vertical line, it is categorized as an *x* error entry. The field of view is divided into appropriately-sized regions, and for measured data points in each region, the *x* and *y* errors are plotted into a histogram for which a distribution is fitted. Assuming a Gaussian distribution, the mean and variance are estimated for the *x* and *y* error of each region.

## 4. Measurement and Results

Depth measurements were taken using the Kinect in accordance with the two scenarios described in Section 3. The sensor characteristics of the Kinect are affected by extreme environmental conditions, such as high temperatures or low lighting, and the device needs several minutes to warm up. Thus, the Kinect is left turned on for several minutes before measurements are taken [14]. To ensure that these environmental factors do not become an additional variable, measurements were taken in a temperature-controlled room with equal light settings and with the Kinect device operating for at least 30 min, so that the device would settle.

### 4.1. Flat Surface Measurement

A flat drywall was selected to be used as a flat planar surface for depth measurements, and a rail perpendicular to the wall was set up. The Kinect sensor was first placed at a distance of 800 mm from the wall on the rail. The Kinect sensor was then adjusted to aim perpendicular to the wall. This was achieved by adjusting the Kinect, so that the depth readings were equal for pixels at similar radial distances from the center.

Once depth measurements were made, the Kinect was moved 200 mm further from the wall along the rail, adjusted to be perpendicular, and a new set of measurements were then taken. Sets of depth images from 800 mm to 3000 mm in 200-mm intervals resulted from this experiment. For each set distance, the Kinect was made to stream depth measurements at 30 frames per second for more than a minute, where 498 of these frames were used to build the model. The original intent was to use 500 samples; however, later, during the post-processing stage, it was revealed that the first and last frames of the recorded 500 samples were corrupted. Thus, the 498 samples were used for the error analysis.

Once distance measurements were made, raw depth readings in (*j_pixel_, i_pixel_, z_mm_*) format were converted into (*x_mm_, y_mm_, z_mm_*) format. The assumption made in this conversion process is that the pixel misalignment is negligible, since actual depth values are similar for neighboring horizontal and vertical locations for a perpendicular flat wall. A plane equation estimation was made based on the *mm* coordinates of the flat surface. After the planer equation was formulated, it was compared against measured points. The result is a statistical distribution of *z* error values for each pixel at each depth interval.

### 4.2. Horizontal and Vertical Measurements

**Figure 7.**
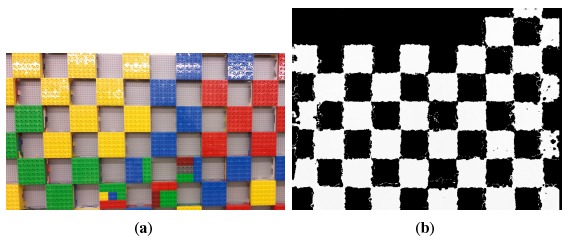
(**a**) The 3D checkerboard used for *x, y* error analysis, and (**b**) the resulting 3D checkerboard measurement.

For the construction of the 3D checkerboard, off-the-shelf building blocks were used. Figure 7a shows the checkerboard, which consists of a checker pattern with a block dimension of 96 mm × 96 mm × 57 mm in the *x-, y*- and *z*-axes, respectively. The checkerboard was mounted on a flat wall, and the Kinect sensor was first placed at a 800-mm distance from the wall. Again, the Kinect was placed along a rail perpendicular to this wall, where the Kinect was adjusted accordingly.

Once depth measurements were made, the Kinect was moved 200 mm further from the wall along the rail, adjusted to be perpendicular, and a new set of measurements were taken. For each distance point, at least 2000 depth image samples were taken, where 498 samples were again used for the error analysis. A sample of this depth measurement is shown in Figure 7b.

Next, distance measurements from the raw depth image in (*j_pixel_, i_pixel_, z_mm_*) format were converted into (*x_mm_, y_mm_, z_mm_*). The *mm* coordinates of the 3D checkerboard edges were used for the checkerboard estimation. The field of view was then divided into 64 equally-sized regions, with each region comprised of 80 × 60 pixels. In the end, means and variances for the distribution in 64 regions for all measured depths were calculated.

### 4.3. Results

**Figure 8.**
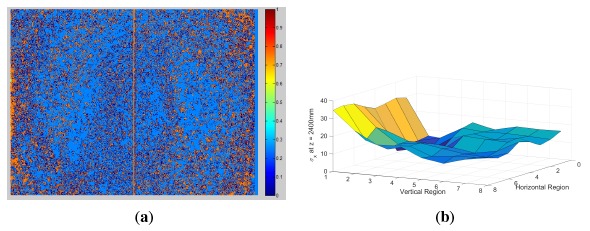
(**a**) The *σ_z_* results at *z* = 1000 mm and (**b**) the *σ_x_* results at *z* = 2400 mm.

The results for *σ_z_* show three trends when inspected visually. First, there is a non-linearly increasing quadratic trend in the *z*-axis, which is present because of the structured light method, as stated in [11]. Next, there is a circular pattern emerging at each *z* slice, with the center located at the center of the depth image originating from the distortion of light as it passed through the circular optical lens. Finally, a lesser, yet noticeable, vertical striping effect occurs. This effect is thought to be caused by the repeating nature of the structured light patterns, but this is yet to be confirmed. For a visualization of the striping effect, Figure 8a shows a border that appears along the edges of the stripes in the *σ_z_* measurement at a distance *z* = 1000 mm.

The results for the *σ_x_* and *σ_y_* error show two trends when inspected visually. First, as with *σ_z_*, there is a quadratically increasing non-linear trend in the *z*-axis, which again is present because of the structured light method [11]. Next, there is a circular pattern emerging at each *z* slice, with the center located at the center of the depth image, which is again caused by lens distortion. An example of the measured results is presented in Figure 8b, in which the *σ_x_* values at *z* = 2400 mm are shown. The major causes of error are the noisy *z* measurements with the addition of the error dependent on the pixel's horizontal and vertical location relative to the center of the field of view.

## 5. Error Model Analysis

### 5.1. Model Construction

Based on the results from Section 4, the model for the standard deviation of error was derived for all three directional error models. All three location variables *i, j* and *z* were chosen as input variables to a quadratic model with the main effects and interaction terms as described in:

(7) $$\sigma \left(i,j,z\right)=\beta_{1}j^{2}+\beta_{2}i^{2}+\beta_{3}z^{2}+\beta_{4}ji+\beta_{5}iz+\beta_{6}zj+\beta_{7}j+\beta_{8}i+\beta_{9}z+\beta_{10}$$

The coefficients *β* were fitted to the measured data based on a least squares approach, as shown in Table 1, where the ten *β* coefficients for each of the *x, y* and *z* models are listed. The difference in coefficients between the *x* and *y* model indicate that a separate model for the horizontal and vertical direction is necessary. Note that the index and depth values are not normalized, where index values range between one and 480 or 640 and depth values are in *mm* starting from 800. Thus, the coefficients of the terms containing depth variables tend to have a lower order of magnitude.

For *σ_x_* and *σ_y_*, it is difficult to see the striping effect, as the errors are grouped into regions that are much more coarse than the stripes. Furthermore, since the error is considered to be equal in each region, the pixel location is translated into region indices as denoted by:

(8) $$\begin{array}{cc} i_{r}=\left\lceil \frac{i}{N_{v}/N_{vr}}\right\rceil & j_{r}=\left\lceil \frac{j}{N_{h}/N_{hr}}\right\rceil \end{array}$$

where *N_h_* and *N_v_* represent the number of horizontal and vertical pixels, respectively, which in the case of the Kinect are 640 and 480. *N_hr_* and *N_vr_* are the number of regions that the field of view is divided into in the horizontal and vertical direction, which, in the case of this paper, is eight for both.

### 5.2. Model Performance

**Figure 9.**
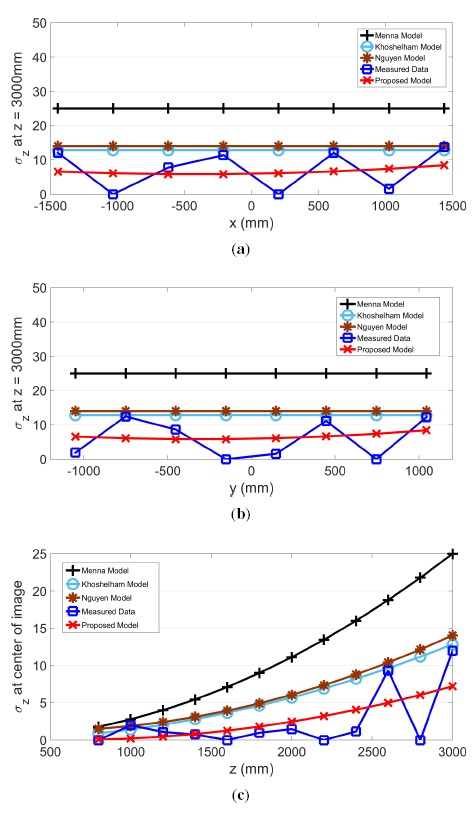
(**a**) shows *σ_z_* along the *x*-axis, and (**b**) and (**c**) show *σ_z_* along the *y* and *z*-axes, respectively.

**Figure 10.**
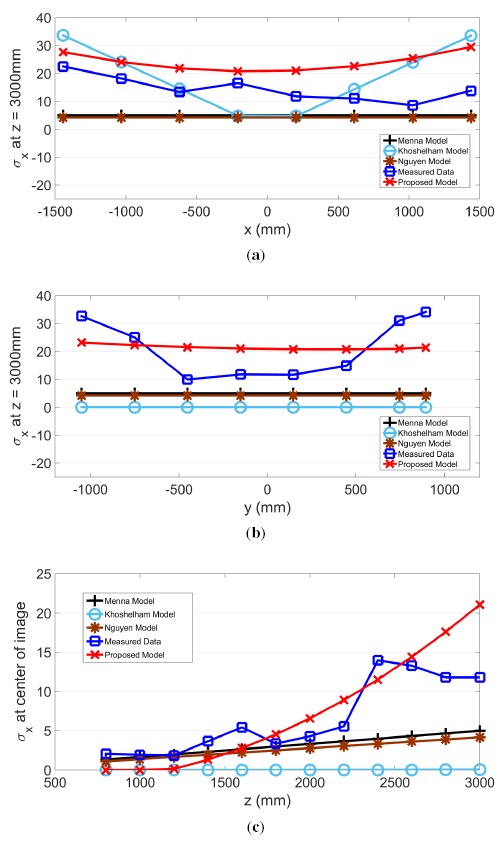
(**a**) *σ_x_* along the *x*-axis and (**b,c**) *σ_x_* along the *y*- and *z*-axes, respectively.

**Figure 11.**
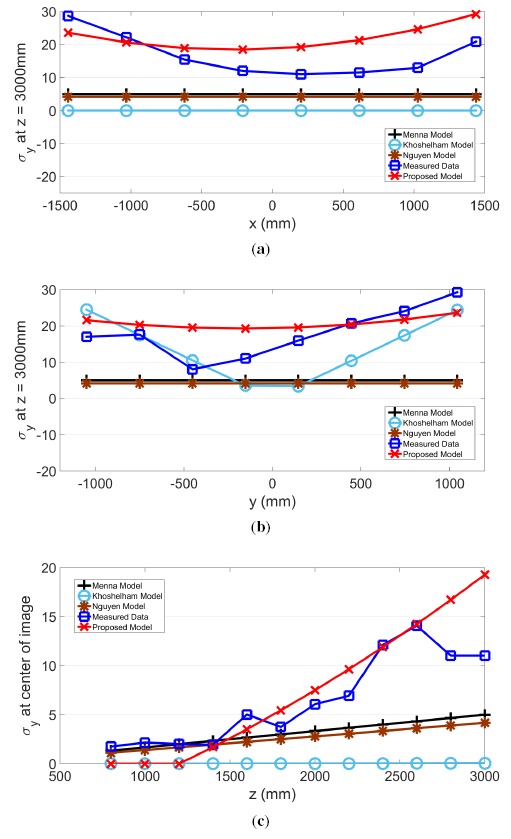
(a) *σ_y_* along the *x*-axis and (**b,c**) *σ_y_* along the *y*- and *z*-axes, respectively.

The proposed model for *σ_z_, σ_x_* and *σ_y_* and actual measurements are plotted in the same graph along the slice of each axis in Figures 9, 10 and 11, respectively. Further plotted in the graphs are three existing models, as proposed by Menna [14], Khoshelham [11] and Nguyen [21]. In the *x* and *y* directions, these three existing models have a stationary value. In other words, values from these models do not change according to the pixel's horizontal or vertical location. However, the actual measured results show that values in *σ_z_, σ_x_* and *σ_y_* change according to the location within the field of view.

The residual sum of squares (*RSS*) value was calculated and compared for all four models. Except for the case of *σ_x_* examined along the *x*-axis, where the proposed model generated the second lowest value, the proposed model shows the best *RSS* results. Therefore, the *RSS* values for the four models shown in Table 2 indicate that the proposed model has the best fit. Furthermore, the proposed model is the only model that performs consistently across all three directions.

In Section 2, several causes for noise were mentioned. Thermal fluctuation, changes in supply power voltage and flickering of the ambient light may all be contributing factors to depth image noise. Thus, the Kinect sensor produces depth estimates in each pixel with an accuracy that partially depends on the recorded intensity of the IR dot patterns that is corrupted by noise. The radial trend in the *x* and *y* directions confirms the lens distortion mentioned in [11], which is accounted for in the proposed model. Another source for the trend is the projector-camera angle, since points with equal projector-camera angles do not move along the depth direction in a linear manner. Though IR dot patterns are used to find a correspondence between projector and camera move in a linear manner, the angle between the camera and projector changes non-linearly. The quantization of the depth slices used to speed up the depth determining process of the Kinect, as mentioned in [15], may also end up as quantization error in the final depth measurement. Moreover, when pixel-pixel-depth measurements are transformed into 3D measurements, the in-plane/lateral values have errors due to quantization and pixelization. These errors are unavoidable yet quantifiable, since we know the size and resolution of each pixel in the depth image. Therefore, the errors in the depth observations occur from a combination of unpredictable noise and systematic error due to distortion, quantization and the conversion of angle to distance by virtue of triangulation.

### 5.3. Pixel Correlation Analysis and Unit Variance

A pixel-to-pixel correlation analysis was performed in order to determine whether there is any correlation between the error in one pixel location and the error of other locations. A collection of 1/16 of *σ_z_* locations was selected at a fixed depth *z* of 1400 mm and compared to itself for a duration of 498 consecutive frames. Results show that there is no significant correlation for *σ_z_* between neighboring pixels. Only eight pixels out of 19, 200 pixels showed a correlation of 0.4 or higher. This confirms the assumption that the error for each pixel is independent.

Since there may be slight deviations in the construction of each Kinect sensor, there is an importance in the stability and repeatability for the proposed error model. In other words, given a different Kinect sensor, the constructed model must be sufficient and accurately characterize the new sensor's error distribution. To demonstrate the similarity of depth images between Kinect units, a second Kinect unit was set up under the same conditions as the primary configuration, and the flat surface scenario measurements were taken for six sample distances ranging from 1000 mm to 3000 mm at 400-mm intervals. The standard deviations for 500 samples were then compared between the two Kinect units. Out of the 640 × 480 × 6 measured points, the number of points with less than 5% difference between the two units were enumerated. Results show that for 99.7% of the points, the difference was less than 5%. This provides evidence that the proposed model is valid and applicable for all Kinect sensors used for research.

## 6. Conclusion

The error characteristics for each of the three dimensions, *i.e.,* the depth (*z*), horizontal (*x*) and vertical (*y*) axes, were measured in this paper. For the actual measurements, a flat surface and a novel method using a 3D checkerboard were presented. The results show that for all three axes, the error should be considered independently. Results also indicate that the distance from the Kinect to the object and the pixel location relative to the center of the image are important elements to include in the proposed error models. The stochastic error models for each of the three axes based on empirical data with pixel location and measured depth as its input variables were also presented. The proposed models were then compared to other existing models, where the proposed models were demonstrated to have an overall better fit to the actual measured data sets. Finally, evidence showing that the model is valid for all Kinect devices was presented.

The results of this paper may be helpful in the construction of a Kinect simulator and in applications that use the Kinect device as a measurement tool for object recognition and tracking, pose estimation and a variety of other uses in research. Furthermore, the method presented in this paper may be applied to other structured light-based depth sensors where a known error model is needed.

## Figures and Tables

**Table 1.** Coefficients for the three directional models.

	***x***	***y***	***z***
*β*_1_	6.3801e-01	6.3038e-01	2.0000e-05
*β*_2_	1.1225e-01	2.6496e-01	2.0000e-05
*β*_3_	3.5751e-06	1.3279e-06	1.2500e-06
*β*_4_	−4.0645e-03	1.5000e-02	2.0000e-06
*β*_5_	−1.4951e-04	9.0174e-05	3.5000e-09
*β*_6_	7.0336e-05	3.3417e-04	3.5000e-09
*β*_7_	−5.6762e+00	−5.9320e+00	−1.0002e-02
*β*_8_	−8.0153e-01	−2.4411e+00	−1.0002e-02
*β*_9_	−3.1496e-03	3.1239e-03	−1.5025e-03
*β*_10_	1.2996e+01	1.0995e+01	1.4515e+00

**Table 2.** *RSS* measurements for the four models.

	***σ****_x_***in**	***σ****_x_***in**	***σ****_x_***in**	***σ****_y_***in**	***σ****_y_***in**	***σ****_y_***in**	***σ****_z_***in**	***σ****_z_***in**	***σ****_z_***in**
	***x* Axis**	***y* Axis**	***z* Axis**	***x* Axis**	***y* Axis**	***z* Axis**	***x* Axis**	***y* Axis**	***z* Axis**
Menna	852.35	2,910.2	291.43	1,412	1,668.5	262.33	61,608	3,110.00	1376.10
Khoshelham	980.38	4,422.5	760.54	2,560	449.3	722.84	11,467	594.82	273.58
Nguyen	987.18	3,131.0	350.56	1,573	1,844.1	320.01	13,216	739.81	327.09
Proposed	900.82	701.12	167.88	453	278.4	127.18	6,962	214.61	103.74

## References

[1] Rude D., Adams S., Cogill R., Beling P. (2014). Task Recognition from Joint Tracking Data Using Simultaneous Feature Selection and Parameter Estimation in Hidden Markov Models.

[2] Cho K.B., Lee B.H. (2012). Intelligent Lead: A Novel HRI Sensor for Guide Robots. Sensors.

[3] Susperregi L., Sierra B., Castrilln M., Lorenzo J., Martínez-Otzeta J., Lazkano E. (2013). On the Use of a Low-Cost Thermal Sensor to Improve Kinect People Detection in a Mobile Robot. Sensors.

[4] Nock C., Taugourdeau O., Delagrange S., Messier C. (2013). Assessing the Potential of Low-Cost 3D Cameras for the Rapid Measurement of Plant Woody Structure. Sensors.

[5] Azzari G., Goulden M., Rusu R. (2013). Rapid Characterization of Vegetation Structure with a Microsoft Kinect Sensor. Sensors.

[6] Palacios J., Sags C., Montijano E., Llorente S. (2013). Human-Computer Interaction Based on Hand Gestures Using RGB-D Sensors. Sensors.

[7] Yang M.T., Chuang M.W. (2013). Fall Risk Assessment and Early-Warning for Toddler Behaviors at Home. Sensors.

[8] Zhou X. (2008). Statistical Model-Based Object Recognition from Three-Dimensional Point-Cloud Data. Ph.D. Thesis.

[9] Reyes I.O., DeVore M.D., Beling P.A., Horowitz B.M. (2010). A probability of error-constrained sequential decision algorithm for data-rich automatic target recognition. Proc. SPIE.

[10] Landau M., DeVore M.D., Beling P.A. Efficacy of Statistical Model-Based Pose Estimation of Rigid Objects with Corresponding CAD Models using Commodity Depth Sensors.

[11] Khoshelham K., Elberink S.O. (2012). Accuracy and Resolution of Kinect Depth Data for Indoor Mapping Applications. Sensors.

[12] Maimone A., Fuchs H. Encumbrance-free telepresence system with real-time 3D capture and display using commodity depth cameras.

[13] Freedman (2010). Depth Mapping Using Projected Patterns.

[14] Menna F., Remondino F., Battisti R., Nocerino E. (2011). Geometric investigation of a gaming active device. Proc. SPIE.

[15] Chow J.C.K., Lichti D.D. (2013). Photogrammetric Bundle Adjustment with Self-Calibration of the PrimeSense 3D Camera Technology: Microsoft Kinect. IEEE Access.

[16] Miller S., Teichman A., Thrun S. Unsupervised extrinsic calibration of depth sensors in dynamic scenes.

[17] Zhang C., Zhang Z. Calibration between depth and color sensors for commodity depth cameras.

[18] Herrera C.D., Kannala J., Heikkila J. (2012). Joint Depth and Color Camera Calibration with Distortion Correction. IEEE Trans. Pattern Anal. Mach. Intell..

[19] Bedok E. (2009). 3D Vision by Using Calibration Pattern with Inertial Sensor and RBF Neural Networks. Sensors.

[20] VDI/VDE (2012). VDI/VDE 2634 Optical 3-D measuring systems.

[21] Nguyen C.V., Izadi S., Lovell D. Modeling Kinect Sensor Noise for Improved 3D Reconstruction and Tracking.

[22] Huddleston S.H., Zhou X., Evans W.B., Chan A., DeVore M.D. (2007). Statistical models for target detection in infrared imagery. Proc. SPIE.

[23] Microsoft Corporation (2013). Kinect for Windows Human Interface Guidelines v1.7.0.

